# *Bambusae caulis* in Liquamen Suppresses the Expression of Thymus and Activation-Regulated Chemokine and Macrophage-Derived Chemokine in Human Keratinocytes due to Antioxidant Effect

**DOI:** 10.1155/2012/617494

**Published:** 2011-07-06

**Authors:** Xu-Feng Qi, Dong-Heui Kim, Yang-Suk Yoon, Soon-Bong Song, Yung-Chien Teng, Dong-Qing Cai, Kyu-Jae Lee

**Affiliations:** ^1^Key Laboratory for Regenerative Medicine, Ministry of Education, Jinan University, Guangzhou 510632, China; ^2^Department of Environmental Medical Biology, Yonsei University Wonju College of Medicine, Wonju, Gangwon 220-701, Republic of Korea; ^3^Institute for Poverty Alleviation and International Development, Yonsei University Wonju Campus, Wonju, Gangwon 220-710, Republic of Korea

## Abstract

*Bambusae caulis* in Liquamen (BCL), traditional herbal medicine used in East Asia, is known to have antioxidative and immune-regulating properties. We hypothesized that the potential antioxidant effects of BCL might suppress the production of thymus and activation-regulated chemokine (TARC) and macrophage-derived chemokine (MDC) in human keratinocytes (HaCaT cell). The immune-regulating effect of BCL was demonstrated by antioxidant capacity using DPPH analysis and DCFH-DA analysis. We found that BCL had strong ROS scavenge effect in HaCaT cell. BCL also showed suppression of IFN-*γ*-induced expression of TARC and MDC, activation of NF-*κ*B, and, moreover, significant block of IFN-*γ*-induced degradation and phosphorylation of I*κ*B. However, it had no effects on phosphorylation of p38 MAPK. Collectively, these results suggest that BCL may have a therapeutic potential on skin disease such as atopic dermatitis by inhibiting Th2 chemokines which is due, at least in part, to its antioxidant capacities.

## 1. Introduction

Chemokines are a superfamily of small cytokines that regulate trafficking of various types of leukocytes [1]. According to the position of the first two of the four conserved cysteine residues, chemokines are divided into four subgroups: CC, C, CXC, and CX3C [2]. Chemokines bind to leukocytes via their corresponding seven transmembrane-spanning, G-protein coupled receptors that have been grouped according to the structure of their chemokine ligands (CCR, CR, CXCR, and CX3CR) [3]. 

Thymus and activation-regulated chemokine (TARC/CCL17) are a CC chemokine constitutively expressed in a thymus and is produced by monocyte-derived dendritic cells [4] and keratinocytes [5]. It is a ligand for CCR4, which is preferentially expressed on Th2 lymphocytes [6]. Macrophage-derived chemokine (MDC/CCL22), a CC chemokine closely related to TARC, is also specific ligand for CCR4. MDC is constitutively produced by dendritic cells, B cells, macrophages, keratinocytes, and epithelial cells [7–9]. TARC and MDC are thought to play an important role in the development of some skin disease such as AD. Previous researchers have reported that the serum levels of TARC and MDC are significantly elevated and correlated with the disease severity in patients with AD [10]. In addition, the expressions of TARC [11] and MDC [5] were significantly increased in the basal epidermis of lesional skin compared with the healthy controls. Taken together, TARC and MDC may be involved in the pathogenesis of AD. 

So far, herbal medicines such as *Rumex japonicus* [12] and *Scutellaria baicalensis* [13] are used to treat atopic dermatitis in Eastern countries. *Bambusae caulis* in Liquamen (BCL), the nutritious liquid that is extracted from heat-treated fresh bamboo stems, is a traditional herbal medicine widely used in Eastern counties for treatment of coughs and asthma. In recent years, it was studied for its anti-inflammatory, antiallergenic, immune-regulating, and antioxidative capacities [14, 15]. Moreover, the liquid diluted with water is gaining widespread popularity in Japan as a folk medicine for skin diseases such as scabies, eczema, and atopic dermatitis [16]. Recently, our study has demonstrated that BCL effectively suppresses the development of 2,4-dinitrochlorobenzene- (DNCB-)induced AD-like skin lesions in hairless mice. Of note, BCL has been shown to regulate the expression of Th2 cytokines such as IL-4 and IL-13 in hairless mice spleen [17]. Furthermore, intracellular reactive oxygen species (ROS) contribute to the production of TARC and MDC in keratinocytes [18]. However, the effects of BCL on the expression of Th2 chemokines in keratinocytes and the potential mechanism have not been evaluated. 

In the present study, we demonstrated that BCL suppresses the expression of TARC and MDC, at least in part, by inhibiting the activation of ROS/IkB/NF-*κ*B signaling pathways independent of p38 MAPK pathway in IFN-*γ*-treated HaCaT cells.

## 2. Materials and Methods

### 2.1. Reagents

*Bambusae caulis* in Liquamen (BCL) was used as described previously [17]. Bay11-7082 and SB203580 ware purchased from Calbiochem (La Jolla, Calif, USA). Hydrogenperoxide (H_2_O_2_), N-acetyl-Leu-Leu-norleucinal (ALLN), 4′,6-Diamidino-2-phenylindole (DAPI), 2,2-diphenyl-1-picrylhydrazyl (DPPH), and 2′,7′-dichlorodihydrofluorescin diacetate (DCFH-DA) were purchased from Sigma-Aldrich Co. (St. Louis, Mo, USA). Recombinant human interferon (IFN)-*γ* was purchased from Abcam (Cambridge, UK). Human TARC/CCL17 and MDC/CCL22 immunoassay kit were purchased from R & D Systems (Minneapolis, Minn, USA). Antibodies against phospho-p38 MAPK, p38 MAPK, NF-*κ*B p65, I*κ*B-*α*, phosphor-I*κ*B-*α*, poly(ADP-ribose) polymerase (PARP), *β*-actin, horseradish peroxidase- (HRP-)conjugated goat anti-rabbit IgG, and Alexa Fluor 488 goat antirabbit IgG (H+L) were purchased from Cell Signaling Technology (Beverly, Mass, USA). An ECL (enhanced chemiluminescence) Western blotting detection system was obtained from Amersham Pharmacia Biotech (Tokyo, Japan). 

### 2.2. Cell Culture

 The human keratinocyte cell line, HaCaT cell, was cultured with RPMI 1640 (BioWhittaker Inc., Walkersville, Md, USA) supplemented with 10% fetal bovine serum (FBS) (BioWhittaker Inc., Walkersville, Md, USA), 1% penicillin/streptomycin (BioWhittaker Inc., Walkersville, Md, USA) at 37°C in a 5% CO_2_ incubator. Before performing experiments described below, the growth medium was exchanged for serum-free RPMI 1640 medium.

### 2.3. Cell Viability Assay

 Cell viability was assessed by the 3-(4,5-dimethylthiazol-2-yl)-2,5-diphenyl tetrazolium bromide (MTT) assay according to the method described previously [19] with some modifications. Briefly, HaCaT cells were seeded into 96-well microtiter plates at a density of 1 × 10^4^ cells/well (200 *μ*L) and incubated in complete medium. After 24 h, the medium was replaced with serum-free medium containing various concentrations of BCL, with RPMI 1640 as the negative control. After incubating for 24 h, cells were treated with MTT (50 *μ*g/mL) for 4 h. The formazan precipitate was dissolved in 200 *μ*L of dimethyl sulfoxide (DMSO), and the absorbance at 570 nm was detected with an automated microplate reader. The percentage of cell viability was calculated by the following formula: cell viability (%) = (mean absorbency in test wells)/(mean absorbency in control wells) × 100.

### 2.4. Enzyme-Linked Immunosorbent Assay (ELISA)

The HaCaT cells (1×10^6^/well) were seeded into 6-well plates in 1 mL medium with 10% FBS. After reaching confluence, the cells were washed and incubated with 1 mL serum-free medium containing the indicated concentration of IFN-*γ* for 24 h. The culture supernatants were analyzed for TARC and MDC by ELISA (R&D Systems) according to the manufacturer's instructions. In some experiments, the HaCaT cells were incubated with BCL or pharmacological inhibitors together with IFN-*γ*.

### 2.5. Reverse Transcription-Polymerase Chain Reaction (RT-PCR)

Total RNA was isolated by using the TRI reagent (Molecular Research Center Inc., Cincinnati, USA). The quantity and purity of RNA were verified by measuring A260 and A280. cDNA was synthesized from total RNA (2 *μ*g) and oligo d (T)_18_ primers (0.5 *μ*g) using *Prime* RT Premix kit (GeNet Bio, Korea) according to the manufacturer's instructions. The cDNA obtained was then amplified using *Prime Taq* Premix kit (GeNet Bio, Korea) following the manufacturer's instruction. The primers used in this study were as follows: TARC (forward) 5′-ATG GCC CCA CTG AAG ATG CT-3′, (reverse) 5′-TGA ACA CCA ACG GTG GAG GT-3′; MDC (forward) 5′-AGG ACA GAG CAT GGC TCG CCT ACA GA-3′, (reverse) 5′-TAA TGG CAG GGA GGT AGG GCT CCT GA-3′; and GAPDH (forward) 5′-ACC ACA GTC CAT GCC ATC AC-3′, (reverse) 5′-TCC ACC ACC CTG TTG CTG TA-3′. GAPDH primers were used as an internal control. All mixtures were denatured at 94°C for 5 min. Conditions of PCR amplification were as follows: TARC, 94°C for 30 s, 62°C for 30 s, and 72°C for 30 s for a total of 30 cycles; MDC, 94°C for 30 s, 65°C for 30 s, and 72°C for 30 s for a total of 32 cycles; GAPDH, 94°C for 30 s, 56°C for 30 s, and 72°C for 30 s for a total of 30 cycles. Following these cycles of PCR amplifications, the amplified cDNAs were further extended by an additional extension at 72°C for 7 min. Amplified products were subjected to electrophoresis on 2% agarose gels and visualized by staining with ethidium bromide.

### 2.6. Whole-Cell and Nuclear Fractionation

The preparation of whole-cell and nuclear extracts were performed using a Nuclear Extract Kit (Active Motif, Carlsbad, Calif, USA). Briefly, HaCaT cells (2 × 10^7^) were washed twice with 3 mL ice-cold PBS (phosphate buffered solution) containing phosphatase inhibitors, centrifuged 5 min at 500 ×g at 4°C, lysed in 300 *μ*L Complete Lysis Buffer, mixed completely and centrifuged at 14 000 ×g for 20 min at 4°C, supernatant (whole-cell extract) were then stored at −80°C until further use. For the nuclear extracts, after washing, cells were lysed in 500 *μ*L hypotonic buffer and then centrifuged at 14 000 ×g for 30 s at 4°C. After removing the supernatant, pellets were resuspended in 50 *μ*L complete lysis buffer and centrifuged at 14 000 ×g for 10 min at 4°C. Supernatants (nuclear fraction) were then stored at −80°C until further use. Protein concentrations were determined using the Bio-Rad Protein Assay (Bio-Rad Laboratories, Calif, USA).

### 2.7. Western Blotting Analysis

 HaCaT cells were treated with medium alone or with IFN-*γ* in the presence or absence of drugs for the indicated time. Proteins (30 *μ*g) were separated on SDS-PAGE and transferred onto nitrocellulose membranes. Membranes were then blocked with 5% nonfat milk, washed briefly, incubated with primary antibodies at 4°C overnight, and then incubated with corresponding HRP-conjugated secondary antibodies for 1 h at room temperature. Protein bands were visualized by incubating membranes with chemiluminescence reagents before exposure to X-ray film. The quantitation of the chemiluminescent signal was analyzed using Image-Pro Plus version 6.0 (Media Cybernetics, Md, USA).

### 2.8. Determination of ROS Generation

The level of intracellular ROS was assessed by measuring the oxidation of 2′,7′-dichlorodihydrofluorescin diacetate (DCFH-DA) using DTX-880 multimode microplate reader (Beckman Coulter Inc., Fullerton, Calif, USA) and fluorescence microscopy (Olympus, Japan) as described previously [18].

### 2.9. Immunocytochemistry

 Immunocytochemistry was performed as described previously [18]. In brief, cells incubated on BD FalconTM culture slides (BD Biosciences, Bedford, Mass, USA) were fixed with 3.7% formaldehyde and permeabilized with 0.5% Triton X-100. Slides were washed with PBS, blocked for one hour with 3% bovine serum albumin, and incubated with rabbit monoclonal anti-NF-*κ*B p65 antibody (1 : 50) overnight at 4°C. The cells were then washed and incubated with Alexa Fluor 488 goat anti-rabbit IgG (H+L) (1 : 200) for one hour at room temperature, and nuclei were stained for 10 min with DAPI (10 *μ*g/mL). The stained cells were mounted with mounting medium and observed by Leica DMI 4000 confocal microscope (Leica, Wetzlar, Germany).

### 2.10. Statistical Analysis

 All data are presented as the mean ± SEM of at least three separate experiments. Statistical analysis was performed using one-way ANOVA followed by Dunnett's multiple comparison test. A *P *value of less than 0.05 was considered to be statistically significant. 

## 3. Results

### 3.1. Cell Viability

**Figure 1:**
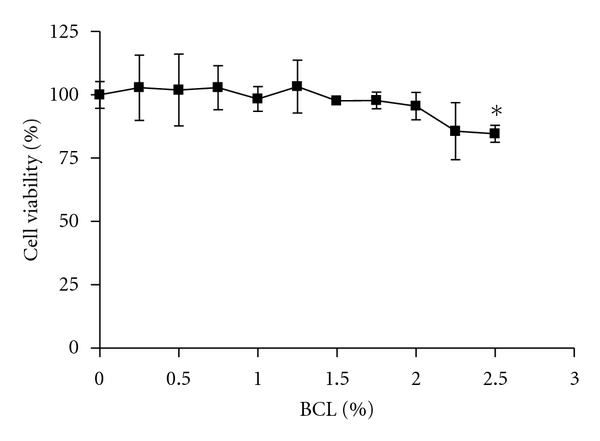
Effect of BCL (*Bambusae caulis *in Liquamen) on the viability of HaCaT cells. All values are presented as mean ± SEM of three experiments conducted in duplicate. *P < 0.05  versus control cells incubated with media alone.

As shown in Figure 1, BCL at 0–2% (v/v) had a minimal effect on the viability of HaCaT cells, although it significantly reduced the viability at the higher concentration of 2.5% after 24 h incubation. Therefore, 2% was determined as the maximum concentration of BCL for use throughout the following experiments. 

### 3.2. BCL Inhibits IFN-*γ*-Induced Expression of TARC and MDC in HaCaT Cells

**Figure 2:**
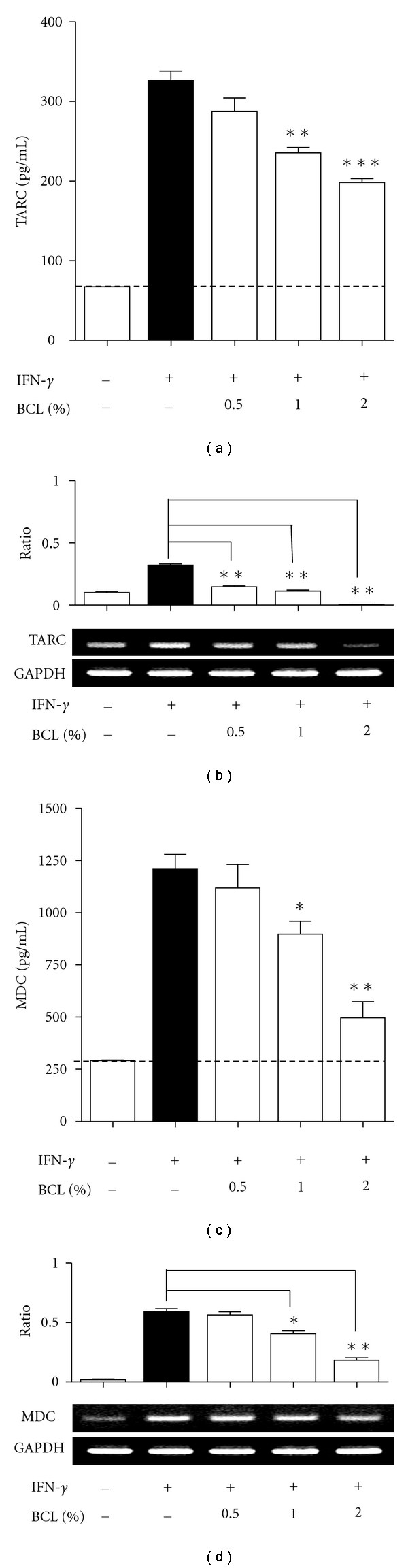
Effects of BCL on IFN-*γ*-induced production of thymus and activation-regulated chemokine (TARC) and macrophage-derived chemokine (MDC) in HaCaT cells. (a) and (b) BCL (0.5%–2%) suppressed IFN-*γ*-stimulated TARC release in a dose-dependent manner. (c) and (d) MDC expression was significantly suppressed at both transcriptional and posttranscriptional levels with the treatment of BCL Data are presented as mean ± SEM of three experiments conducted in duplicate. *P < 0.05, **P < 0.01, and ***P < 0.001  versus IFN-*γ*.

 BCL (0.5%–2%) suppressed IFN-*γ*-stimulated TARC release in a dose-dependent manner (Figure 2(a)). IFN-*γ* greatly induced TARC release (326.6 ± 11.20 pg/mL) from HaCaT cells, and this release was reduced to 235.3 ± 6.948 pg/mL (*P* < 0.01) and 198.2 ± 4.977 pg/mL (*P* < 0.001) by treatment of BCL at 1% and 2%, respectively. Similarly, BCL dose-dependently suppressed IFN-*γ*-stimulated mRNA expression of TARC (Figure 2(b)). In a way similar to TARC, MDC expression was significantly suppressed at both transcriptional and posttranscriptional levels with the treatment of BCL (Figures 2(c) and 2(d)). 

### 3.3. BCL Inhibits the Activation of NF-*κ*B but Not p38 MAPK in HaCaT Cells

**Figure 3:**
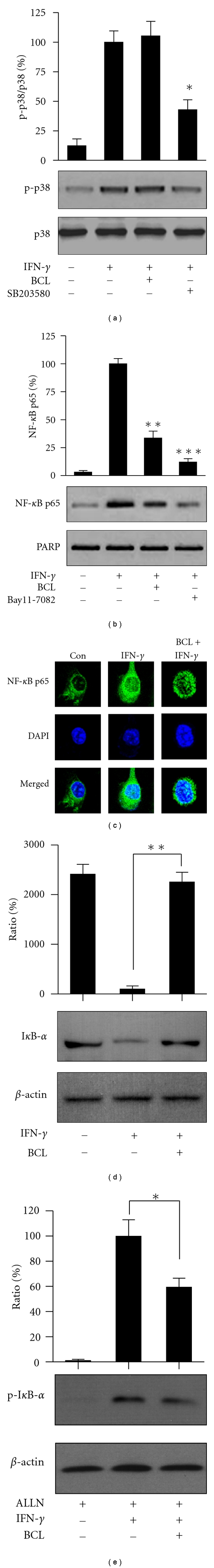
BCL suppressed the activation of NF-*κ*B via I*κ*B but not via p38 pathway. The phosphorylation of p38 MAPK induced after incubation with IFN-*γ* was not affected by BCL (2%) treatment. Phosphorylation of p38 MAPK was significantly suppressed by SB203580. (b) IFN-*γ* significantly increased the nuclear translocation of NF-*κ*B p65, but BCL (2%) decreased the nuclear levels of NF-*κ*B p65 by about 60% in IFN-*γ*-stimulated HaCaT cells. NF-*κ*B inhibitor, Bay11-7082, suppressed IFN-*γ*-induced nuclear translocation of NF-*κ*B by about 85%. (c) The inhibitory effect of BCL on IFN-*γ*-induced nuclear translocation of NF-*κ*B p65 was observed using immunocytochemistry analysis. (d) BCL (2%) markedly blocked IFN-*γ*- (10 ng/mL)induced degradation of I*κ*B-*α*. (e) IFN-*γ*-induced phosphorylation of I*κ*B-*α* was also significantly suppressed by the addition of 2% BCL. All of data are presented as mean ± SEM of three separate experiments. *P < 0.05, **P < 0.01, and ***P < 0.001  versus IFN-*γ* alone.

The activation of p38 MAPK results in phosphorylation at threonine and tyrosine residues of this kinase [20]. In this study, the phosphorylation of p38 MAPK was induced following 30 min incubation with IFN-*γ* (10 ng/mL), and this phosphorylation was not affected by BCL (2%) treatment. However, the specific inhibitor of p38 MAPK, SB203580, significantly suppressed the phosphorylation of p38 MAPK (Figure 3(a)). 

On the other hand, the p65 subunit is the primary component of activated NF-*κ*B, the activation of NF-*κ*B in IFN-*γ*-stimulated HaCaT cells was studied by measuring the nuclear translocation of NF-*κ*B subunit p65 by Western blot. IFN-*γ* significantly increased the nuclear translocation of NF-*κ*B p65 at 30 min. BCL (2%) decreased the nuclear levels of NF-*κ*B p65 by approximately 60% in IFN-*γ*-stimulated HaCaT cells (*P* < 0.01) (Figure 3(b)), strongly suggesting that BCL can inhibit the activation of NF-*κ*B in HaCaT cells. In a manner similar to BCL, the NF-*κ*B inhibitor, Bay11-7082, suppressed IFN-*γ*-induced nuclear translocation of NF-*κ*B by approximately 85% (Figure 3(b)). Furthermore, the inhibitory effect of BCL on IFN-*γ*-induced nuclear translocation of NF-*κ*B p65 was also observed using immunocytochemistry analysis (Figure 3(c)). Collectively, these findings strongly suggest that BCL can inhibit the activation of NF-*κ*B in HaCaT cells. 

### 3.4. BCL Blocked the Degradation and Phosphorylation of I*κ*B in HaCaT Cells

In our Western blotting results, BCL (2%) markedly blocked IFN-*γ*-(10 ng/mL)induced degradation of I*κ*B-*α* (*P* < 0.01) (Figure 3(d)). In addition, IFN-*γ*-induced phosphorylation of I*κ*B-*α* was also significantly suppressed by the addition of 2% BCL (*P* < 0.05) (Figure 3(e)). Taken together, BCL could suppress the activation of NF-*κ*B through I*κ*B-*α* signaling but not through p38 MAPK signaling in HaCaT cells.

### 3.5. Determination of Antioxidant Effects of BCL

**Figure 4:**
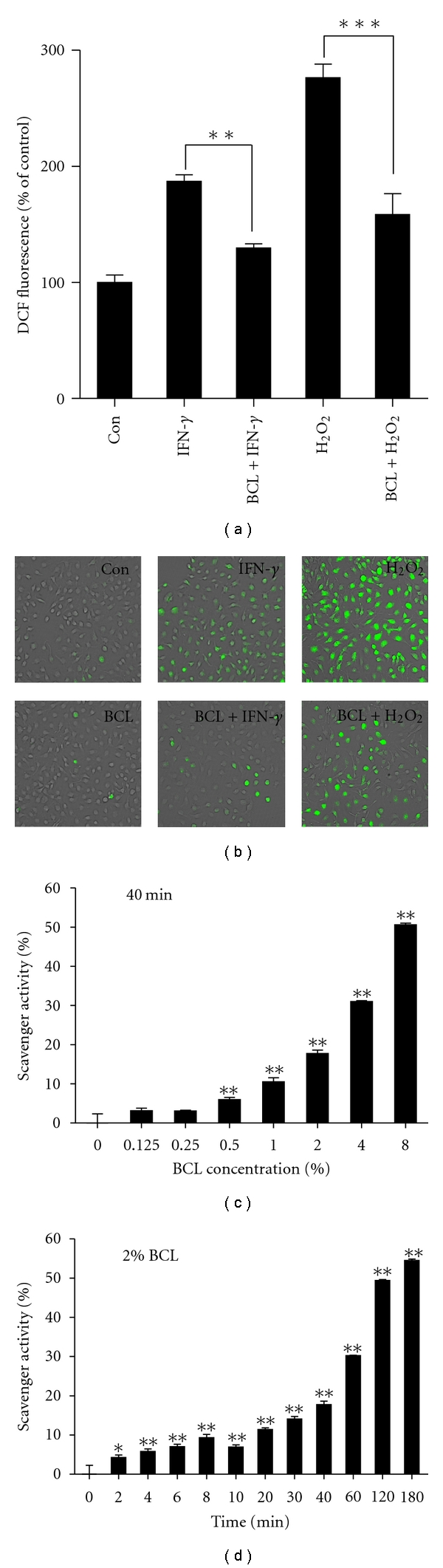
Antioxidant effects of BCL. (a) ROS generation induced by IFN-*γ* or H_2_O_2_ were significantly blocked by the addition of BCL. (b) Typical images of ROS generation (green fluorescence) under the fluorescence microscope (100x). (c) and (d) DPPH free radicals could be significantly scavenged both in dose- and time-dependent manners. Data are presented as the mean ± SEM of three separate experiments conducted in duplicate. *P < 0.05, **P < 0.01, and ***P < 0.001 versus control.

 Intracellular ROS generation contributes to the production of Th2 chemokines by increasing activation of IkB/NF-*κ*B pathways [18]. As the results of DCFH-DA analysis and DPPH analysis using BCL, both ROS generation induced by IFN-*γ* or H_2_O_2_ was significantly blocked by the addition of BCL (*P* < 0.01 and *P* < 0.001, resp.) (Figure 4(a)). Moreover, typical green fluorescence indicative of the generation of ROS was also observed under the fluorescence microscope (Ex = 488 nm, Em = 525 nm, Olympus) (Figure 4(b)), indicating that BCL possesses the ability to scavenge ROS in human keratinocytes. In addition, DPPH analysis showed that DPPH free radicals could be significantly scavenged both in dose- and time-dependent manners (Figures 4(c) and 4(d)). These results strongly suggest that BCL possesses antioxidant effects, thereby suppressing the production of Th2 chemokines via blocking ROS/I*κ*B/NF-*κ*B pathways in HaCaT cells. 

## 4. Discussion

In the present study, we investigated the *in vitro* effects of BCL on Th2-type immune response using HaCaT cells, an *in vitro* model of atopic dermatitis [21]. Our results clearly showed that BCL suppressed the expression of TARC and MDC in HaCaT cells, which strongly indicates that BCL might be used clinically as a potential therapeutic agent for atopic dermatitis. 

Atopic dermatitis is a Th2-type skin disease in which the inflammation is characterized by the infiltration of lymphocytes into a dermis. TARC is one of the functional ligands for CCR4 which is preferentially expressed on Th2-type cells, and chemoattracts the CCR4-Th2-type cells into lesional sites in skin [8, 9]. Previous studies have demonstrated that TARC is highly expressed in a basal epidermis of lesional skin in NC/Nga mice [5] and in different layers of lesional epidermis from patients with AD but not in the nonlesional skin [11]. MDC is another important chemokine for lymphocytes infiltration in inflammatory sites, and its receptor is CCR4 as TARC. High level of MDC was observed in the monocyte-derived dendritic cells (MoDCs) isolated from venous blood in patients with AD [22] and in keratinocytes in lesional skin of atopic dermatitis [23]. Taken together, the levels of TARC and MDC were significantly higher in AD patients compared with the healthy control subjects [10]. These preclinical and clinical studies suggest that TARC and MDC are major molecules which are involved in the pathogenesis of AD. Interestingly, our results show that BCL suppresses IFN-*γ*-induced expression of TARC and MDC in human HaCaT keratinocytes, indicating that BCL possesses immunosuppressive properties. This hypothesis is consistent with previous studies showing that BCL suppressed the expression of different CD (cluster of differentiation) genes in human mast cells [14] and that BCL is used as a folk medicine for skin diseases, including eczema and atopic dermatitis [16]. Furthermore, BCL inhibits the development of DNCB-induced AD-like skin lesions in hairless mice by suppressing Th2 immune response [17]. 

Previous studies have demonstrated that several signaling molecules such as JAKs, MAPKs, STAT, or NF-*κ*B are involved in the regulation of immune responses depending on the cell type [24–27]. Furthermore, both promoters of TARC and MDC contain STAT- and NF-*κ*B-binding sites [26], and the transcriptional activities of STAT1 and NF-*κ*B are increased in keratinocytes with treatment of IFN-*γ* [28], so these transcription factors may be involved in the mediation of these two chemokines. Our recent study found that both p38 MAPK and ROS signaling contribute the activation of NF-*κ*B pathway, respectively [18]. Although p38 MAPK contributes to the activation of NF-*κ*B in keratinocytes [25, 29], the present study showed that BCL significantly suppressed the nuclear translocation of NF-*κ*B, but not phosphorylation of p38 MAPK, suggesting that BCL inhibits the activation of NF-*κ*B but not that of p38 MAPK. This idea is further supported by several previous studies showing that certain bamboo extracts inhibit IL-12 in mouse macrophages via suppression of NF-*κ*B inflammatory signaling pathway [30] and that certain herbal extracts such as polyphenols and polysaccharides possess immune-regulating or anti-inflammatory properties [31, 32]. 

Reactive oxygen species (ROS) is one of the important biomarkers of oxidative stress, and it can function as second messengers during inflammatory signaling [33] and contribute to proinflammatory effects and nuclear factor-*κ*B activation in immune cells [34] and keratinocytes [18]. IFN-*γ* has been shown to increase ROS generation in cultured human retinal pigment epithelial (RPE) cells [35], mouse primary hepatocytes [36], mouse biliary epithelial cells (BECs) [37], human endothelial cells [38], and human keratinocytes [18]. Taken together, these previous data suggest that intracellular ROS levels are intimately associated with the development of inflammation dependent on cell types. BCL is the nutritious liquid isolated from fresh bamboo stems which contain various biologically active components, such as flavonoid, phenolic compounds, polyphenolic compounds, chlorogenic acid derivatives, and various organic acids. Many of these ingredients have been reported to have antioxidant effect [15, 16, 39, 40]. As expected, we directly demonstrated the antioxidant activity of BCL in this study, indicating that the antioxidant activity of BCL contributes to the inhibition of NF-*κ*B and thereby suppresses the production of TARC and MDC in human keratinocytes.

In conclusion, BCL significantly suppressed IFN-*γ*-induced expression of TARC and MDC by inhibiting the activation of ROS/I*κ*B/NF-*κ*B pathways, in a p38 MAPK-independent manner in HaCaT cells. These results are supposed to be due, at least in part, to its antioxidant effects. The blocking of Th2 chemokines by BCL may suppress the infiltration of Th2 cells and lessen inflammation of skin lesions. It suggests that BCL may have a therapeutic potential on skin disease such as atopic dermatitis by inhibiting Th2 chemokines, which is due, at least in part, to its antioxidant capacities.
